# Retraction Note: Lactate dehydrogenase D serves as a novel biomarker for prognosis and immune infiltration in lung adenocarcinoma

**DOI:** 10.1186/s12885-024-13137-1

**Published:** 2024-11-07

**Authors:** Yu Zhang, Tianyi Zhang, Yingdong Zhao, Hongdi Wu, Qiang Zhen, Suwei Zhu, Shaoshuai Hou

**Affiliations:** 1 Department of Pulmonary and Critical Care Medicine, Shandong Provincial Hospital, Shandong University, Jinan, Shandong 250021 China; 2 Research Center of Translational Medicine, Jinan Central HospitalAffiliated to, Shandong First Medical University, Jinan, Shandong 250021 China; 3 Liaocheng Third People’s Hospital, Liaocheng, Shandong 252000 China; 4 Department of Fundamental, Air Force Communications NCO Academy, Dalian, Liaoning, 116000 China; 5 College of Pharmacy, Shandong First Medical University, Jinan, Shandong 250021 China; 6 Department of Critical-Care Medicine, Shandong Provincial HospitalAffiliated to, Shandong First Medical University, Jinan, Shandong 250021 China; 7 Department of Pharmacy, Tengzhou Central People’s Hospital, Tengzhou, Shandong 277500 China

**Retraction Note** **:** **BMC Cancer 23, 759 (2023)**

**https://doi.org/10.1186/s12885-023-11221-6**

The Editor has retracted this article after concerns were raised about some of the data reported. Specifically:

In Figure 10, the image panels for normal tissue Case 1 and Case 2 appear to overlap, though the two panels are presented with different colouration;

In Figure 11d, the image panels for siNC in H838 cells appear to be duplicates.

The Editor no longer has confidence in the reliability of this article's findings and conclusion.

Shaoshuai Hou disagrees with this retraction. The remaining authors did not respond to correspondence from the Publisher regarding this retraction.

